# Environmental impact of the diet of young Portuguese and its relationship with adherence to the Mediterranean Diet

**DOI:** 10.1007/s00394-024-03396-w

**Published:** 2024-05-19

**Authors:** Laura Álvarez-Álvarez, Facundo Vitelli-Storelli, María Rubín-García, Vicente Martín-Sánchez, Camino García Fernández, Catarina Carvalho, Joana Araújo, Elisabete Ramos

**Affiliations:** 1 Group of Investigation in Interactions Gene-Environment and Health (GIIGAS), Institute of Biomedicine (IBIOMED), University of León, León, Spain; 2 CIBER de Epidemiología y Salud Pública (CIBERESP), Instituto de Salud Carlos III, Madrid, Spain; 3 Department of Food Hygiene and Technology, Veterinary Faculty, University of León, León, Spain; 4 Department of Public Health and Forensic Sciences, EPIUnit-Institute of Public Health, University of Porto, Porto, Portugal; 5 Laboratory for Integrative and Translational Research in Population Health (ITR), University of Porto, Porto, Portugal; 6 Medical School, University of Porto, Porto, Portugal

**Keywords:** Climate change, Environmental sustainability, Healthy dietary pattern, Sustainable food

## Abstract

**Objective:**

To estimate, in a cohort of young Portuguese adults, the environmental impact (greenhouse gas (GHG) emissions, land use, energy used, acidification and potential eutrophication) of diet according to adherence to the Mediterranean Diet (MD).

**Methods:**

Data from 1554 participants of the Epidemiologic Health Investigation of Teenagers in Porto (EPITeen) were analysed. Food intake and MD adherence were determined using validated questionnaires. The environmental impact was evaluated with the EAT-Lancet Commission tables, and the link between MD adherence and environmental impact was calculated using adjusted multivariate linear regression models.

**Results:**

Higher adherence (high vs. low) to the MD was associated with lower environmental impact in terms of land use (7.8 vs. 8.5 m^2^, p = 0.002), potential acidification (57.8 vs. 62.4 g SO2-eq, p = 0.001) and eutrophication (21.7 vs. 23.5 g PO4-eq, p < 0.001). Energy use decreased only in the calorie-adjusted model (9689.5 vs. 10,265.9 kJ, p < 0.001), and GHG emissions were reduced only in a complementary model where fish consumption was eliminated (3035.3 vs. 3281.2 g CO2-eq, p < 0.001). Meat products had the greatest environmental impact for all five environmental factors analysed: 35.7% in GHG emissions, 60.9% in energy use, 72.8% in land use, 70% in acidification and 61.8% in eutrophication.

**Conclusions:**

Higher adherence to the MD is associated with lower environmental impact, particularly in terms of acidification, eutrophication, and land use. Reducing meat consumption can contribute to greater environmental sustainability.

**Supplementary Information:**

The online version contains supplementary material available at 10.1007/s00394-024-03396-w.

## Introduction

Climate change is one of the great problems of the twenty-first century, and there are increasing strategies aimed at reducing the impact of humans on the planet. In this sense, diet plays a key role in climate change as it is considered to be responsible for one-third of greenhouse gas (GHG) emissions [1, 2], two-thirds of fresh water use [3] and the use of about half of the planet’s ice-free surface as farmland or grasslands [4]. This is so important that, even if fossil fuel emissions were halted, GHG emissions from the food system could impede meeting the Paris Climate Agreement's goal of limiting global warming to 1.5 °C compared to pre-industrial levels [5].

Since not all foods have the same environmental impact, different food patterns are particularly relevant in terms of their relationship with environmental sustainability [4, 6, 7]. Today, the concept of sustainable diets is becoming increasingly important. The Food and Agriculture Organization defines sustainable diets as those “with low environmental impacts which contribute to food and nutrition security and to healthy life for present and future generations” with specific features, such as “protective and respectful of biodiversity and ecosystems, culturally acceptable, accessible, economically fair and affordable; nutritionally adequate, safe and healthy; while optimising natural and human resources” [8].

In 2019, the EAT-Lancet Commission published a reinforcing that use dietary patterns more sustainable than current Western patterns, in which consumption of meat and dairy products is reduced, is the most important strategy to improve the sustainability of the current food system [9].

One of the dietary patterns that meets these characteristics is the Mediterranean Diet (MD), characterised by a high intake of vegetable products and monounsaturated fatty acids (mainly olive oil); a moderate intake of fish; a low-moderate intake of meat, poultry and dairy products; and a moderate intake of wine [10]. This dietary pattern is recognised for its important benefits in cardiovascular health [11, 12] and the prevention of chronic diseases [13, 14]. Moreover, since it reduces the intake of animal products and promotes biodiversity [15, 16], this dietary model is expected to benefit environmental sustainability.

Understanding the environmental impact of diets is fundamental for developing sustainable public health policies that contribute to improving global health. Therefore, the aim of this work was to estimate, in a cohort of young Portuguese adults, the environmental impact of diet according to the adherence to the MD.

## Methods

The Epidemiologic Health Investigation of Teenagers in Porto (EPITeen) is a cohort study of adolescents who were born in 1990, and attended public and private schools in Porto, Portugal, in 2003/2004, as previously described [17]. Data from the third wave of the study in 2011/2013, when participants were, on average, 21 years old, were used for the present analysis.

The study was approved by the Ethics Committee of the Hospital São João and the Ethics Committee of the Institute of Public Health of the University of Porto on July 31, 2012. Written informed consent was obtained from parents and participants at baseline, participants in the third wave. In addition, a secure standard procedure was followed to ensure confidentiality and data protection.

### Participants

At 21 years, 1764 participants were evaluated, but after excluding participants without food frequency questionnaire (FFQ) information, those with total energy intake higher than three times the interquartile range, or fruit or vegetable intake more than 1.5 times the interquartile range, and those with extreme total energy intakes (< 500 or > 3500 kcal/day in women or < 800 or > 4000 kcal/day in men) [18], 1554 participants remained for the analysis.

### Variables and data collection

The dietary assessment was carried out using an 86-item semi-quantitative food frequency questionnaire (FFQ) that was validated in the Portuguese adult population [19, 20]. The FFQ covered the previous 12 months, ranging from never or less than once a month to six or more times a day. Daily energy intake and dietary fibre were calculated using Food Processor Plus® software (ESHA Research, Salem, OR, USA), with the addition of values for Portuguese foods based on the Portuguese Food Composition Tables, typical recipes and previous data [19, 21].

Adherence to MD was calculated according to the Dietary Score (DS) index, developed by Panagiotakos [22], which includes 11 food groups (vegetables, legumes, fruits, fish, whole grains, potatoes, olive oil, poultry, dairy products with fat, red meat and alcohol), and ranges from 0 to 55. This index classifies adherence into tertiles, with the first tertile corresponding to low adherence and the third tertile to high adherence.

Trained interviewers used face-to-face interviews and self-administered questionnaires to collect data on sex, body mass index (BMI), parental and own education level, lifestyle, and dietary habits.

The educational level of the parents was obtained from the information of the parent with the most advanced formal education, represented as the number of years of formal education successfully completed. Schools were categorized as public if they were state-run, and as private otherwise. BMI classification was based on the World Health Organization definition [23], while sports activity was evaluated according to the frequency of dedication of at least 20 consecutive minutes to sports activities, excluding compulsory school activities.

### Estimating environmental footprint

Based on the information gathered in the FFQs, GHG emissions (grams of Carbon Dioxide equivalents (g CO2-eq)), land (m2) and energy use (kJ), acidification (grams of Sulfur Dioxide equivalents (g SO2-eq)) and eutrophication potential (grams of Phosphate equivalent (g PO4-eq)) were estimated and associated with each food item. These estimations were based on the EAT-Lancet Commission tables [9], using the following steps:

1. All FFQ food items without reference in the tables of the EAT-Lancet Commission were excluded and reduced to 63 foods (We lack information on chocolate, turkey, certain fruits, vegetables and legumes, and also on certain beverages such as soft drinks, coffee or tea);
2. For dishes and recipes, we calculated their environmental impact based on their ingredients and proportions, using traditional MD recipes [24, 25];
3. When a FFQ item did not refer to a single food (e.g., blue fish), we calculated the intake ratio based on data from the National Food and Physical Activity Survey [26].
4. The environmental loads of each food were obtained from the meta-analysis [27] published within the recommendations of the EAT-Lancet Commission (Supplementary material), and the environmental impact of each food was calculated by multiplying the value of the environmental burden by the daily consumption of each product;
5. Finally, to determine the environmental impact of each participant's diet, we summed up the individual contributions of all the foods consumed considering the information collected in the FFQs.

### Statistical analysis

Descriptive statistics were used to show the general baseline characteristics of the participants. The prevalence for each category of each qualitative variable described is expressed as n and percentage. To test whether there were differences between the groups described, the chi-squared test was used, including its corresponding p value. Dietary intake values for food groups and environmental footprints were represented by means and standard deviations. Linear regression models, adjusted for sex, total energy intake and parents’ years of schooling (≤ 8 years, 9–12 years or ≥ 13 years), were performed to classify participants based on tertiles of adherence to MD, and Kruskal–Wallis tests were used to assess differences between tertiles with respect to GHG emissions, land and energy use, acidification, and eutrophication.

R software version 4.1.1 [28] was used for the calculation of individuals’ dietary environmental impact, and statistical analysis was performed using Stata software version 15.1 [29].

## Results

The sample included 1554 participants of whom 51% were women, 75% had studied in public school, 42% had studied for more than 15 years and 71% of their parents had studied for 9 years or more, 69% had normal weight, 51% did sports, 63% were non-smokers or former smokers and 62% consumed alcohol less than once a week. A higher level of education, both from the participant and from the parents, the practice of sports activities, not smoking and not drinking alcohol were significantly associated (p < 0.05) with greater adherence to MD according to the DS index (Table 1).

**Table 1:** General characteristics of the sample according to tertiles of the Dietary Score (DS)

	Overall (n = 1554)		Tertiles MD			
	n	%	Low (0–28)	Medium (29–33)	High (34–54)	*p-value*
Sex						
Female	800	51.5	288 (49.8%)	270 (50.6%)	242 (54.8%)	0.130
Male	754	48.5	290 (50.2%)	264 (49.4%)	200 (45.3%)	
Type of school^a^						
Public	1032	75.2	388 (76.7%)	355 (75.7%)	289 (72.6%)	
Private	270	19.7	88 (17.4%)	92 (19.6%)	90 (22.6%)	0.124
Professional	71	5.2	30 (5.9%)	22 (4.7%)	19 (4.8%)	
Missing	181					
Parents’ years of schooling						
≤ 8 years	441	28.5	186 (32.4%)	142 (26.8%)	113 (25.6%)	
9–12 years	607	39.3	208 (36.2%)	220 (41.6%)	179 (40.5%)	**0.046**
≥ 13 years	497	32.2	180 (31.4%)	167 (31.6%)	150 (33.9%)	
Missing	9					
Participant’s years of schooling						
≤ 12 years	530	38.0	242 (46.5%)	170 (35.6%)	118 (29.8%)	
13–14 years	283	20.3	98 (18.8%)	107 (22.4%)	78 (19.7%)	** < 0.001**
≥ 15 years	581	41.7	181 (34.7%)	200 (41.9%)	200 (50.5%)	
Missing	160					
BMI (kg/m^2^)						
< 18.5	93	6.0	43 (7.4%)	23 (4.3%)	27 (6.1%)	
≥ 18.5 and < 25	1074	69.1	404 (69.9%)	366 (68.5%)	304 (68.8%)	
≥ 25 and < 30	289	18.6	96 (16.6%)	109 (20.4%)	84 (2%)	0.568
≥ 30	98	6.3	35 (6.1%)	36 (6.7%)	27 (6.1%)	
Sports						
No	758	48.8	308 (53.3%)	251 (47.0%)	199 (45.0%)	
Yes	796	51.2	270 (46.7%)	283 (53.0%)	243 (55.0%)	**0.007**
Smoking habits						
Non-smoker	435	28.0	133 (23.1%)	156 (29.2%)	146 (33.0%)	
Smoker	568	36.6	257 (44.5%)	183 (34.3%)	128 (29.0%)	** < 0.001**
Former smoker	550	35.4	187 (32.4%)	195 (36.5%)	168 (38.0%)	
Missing	1					
Alcohol consumption						
No	39	2.5	17 (2.9%)	10 (1.9%)	12 (2.7%)	
Yes	545	35.1	236 (40.9%)	174 (32.6%)	135 (30.5%)	**0.001**
Former drinker or < 1 time/week	969	62.4	324 (56.2%)	350 (65.5%)	295 (66.8%)	
Missing	1					

The p values correspond to the chi-square test performed to assess whether there are differences between groups. *BMI* Body Mass Index, *MD* Mediterranean Diet

^a^Type of school at age 21, data collected in the third wave of the study

The results highlighted in bold are those statistically significant (p < 0.05)

**Fig. 1:**
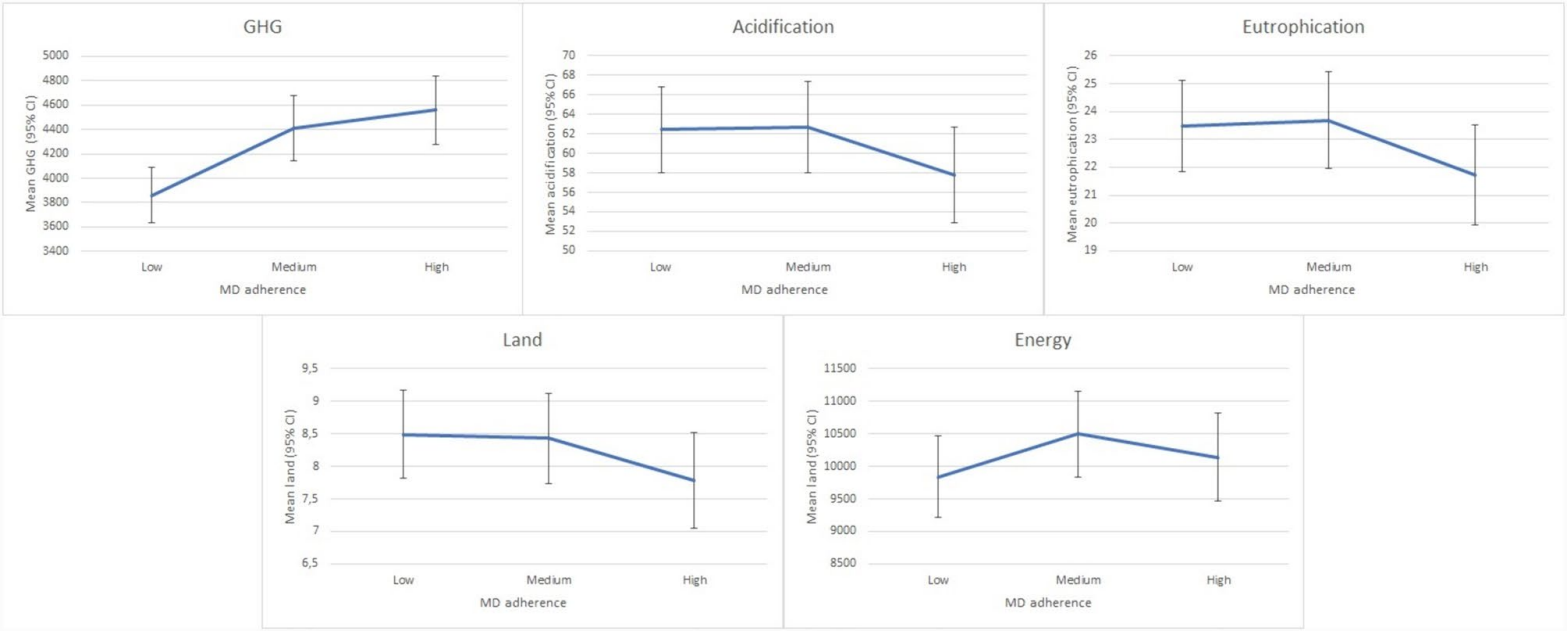
Environmental footprint for different factors by tertiles of adherence to MD (Crude model)

The results obtained in relation to environmental factors according to adherence to the MD dietary pattern by tertiles are shown in Fig. 1. In the crude model, higher adherence to MD was associated with lower acidification (high vs. low adherence: 57.8 vs. 62.4 g SO2-eq, p = 0.001), lower eutrophication (high vs. low adherence: 21.7 vs. 23.5 g PO4-eq, p = 0.001) and lower land use (high vs. low adherence: 7.8 vs. 8.5 m^2^, p = 0.001). However, greater adherence to MD was associated with higher GHG emissions (high vs. low adherence: 4561.7 vs. 3861.6 g CO2-eq, p < 0.001) and increased energy use (high vs. low adherence: 10,140 vs. 9840 kJ, p = 0.144).

Subsequently, different models were estimated, adjusting for total energy intake and then for sex and parents’ years of schooling (Table 2). In the model adjusted for total energy intake, higher adherence to MD was associated with lower acidification (high vs. low adherence: 54.5 vs. 65.5 g SO_2_-eq, p < 0.001), lower eutrophication (high vs. low adherence: 20.5 vs. 24.6 g PO4-eq, p < 0.001), lower land use (high vs. low adherence: 7.3 vs. 8.9 m^2^, p < 0.001) and lower energy use (high vs. low adherence: 9689.5 vs. 10,265.9 kJ, p = 0.001). For GHG emission, we found a lower impact for those with lower adherence to MD (4042.8 g CO2-eq) and similar impact for the second and third tertile (4397.3 and 4370.0 g CO2-eq, p < 0.001, respectively).

**Table 2:** Environmental footprint for different factors by tertiles of the Diet Score (DS) according to different adjustment models

	Low adherence to the MD		Medium adherence to the MD		High adherence to the MD		*p-value*
	Mean	95% CI	Mean	95% CI	Mean	95% CI	
Linear regression model adjusted for total energy intake							
GHG (g CO2-eq)	4042.9	3951.4–4134.3	4397.3	4304.7–4490.0	4370.0	4271.9–4468.2	** < 0.001**
Acidification (g SO2-eq)	65.5	63.9–67.2	62.4	60.8–64.1	54.5	52.7–56.3	** < 0.001**
Eutrophication (g PO4-eq)	24.6	24.0–25.3	23.6	23.0–24.2	20.5	19.9–21.2	** < 0.001**
Land (m2)	8.9	8.7–9.2	8.4	8.1–8.7	7.3	7.1–7.6	** < 0.001**
Eenergy (kJ)	10,265.9	10,030.3–10,501.5	10,461.1	10,222.4–10,699.7	9689.5	9436.7–9942.3	** < 0.001**
Linear regression model adjusted for sex and parents' years of schooling							
GHG (g CO2-eq)	3883.71	3779.2–3988.2	4366.9	4260.0–4473.8	4573.48	4461.6–4685.3	** < 0.001**
Acidification (g SO2-eq)	62.86	61.1–64.7	61.7	59.9–63.5	58.14	56.2–60.1	** < 0.001**
Eutrophication (g PO4-eq)	23.61	22.9–24.3	23.33	22.6–24.0	21.86	21.1–22.6	** < 0.001**
Land (m2)	8.55	8.3–8.8	8.28	8.0–8.6	7.83	7.5–8.1	** < 0.001**
Eenergy (kJ)	9905.92	9648.2–10,163.7	10,363.22	10,099.4–10,627.0	10,169.6	9893.6–10,445.6	** < 0.001**

The table shows the mean adjusted for the different environmental impact factors. The results of the linear regression models were adjusted for total calorie intake in the first case, and for sex and years of parental schooling in the second

*MD* Mediterranean Diet, *GHG* greenhouse gas emissions, *CI* Confidence Interval, *g CO2-eq* grams of Carbon Dioxide equivalents, *g SO2-eq* grams of Sulfur Dioxide equivalents, *g PO4-eq* grams of Phosphate equivalents, *kJ* kilojoules

The results highlighted in bold are those statistically significant (p < 0.05)

In the model adjusted for sex and parents’ years of schooling, we found that greater adherence to MD was significantly associated with lower acidification (high vs. low adherence: 58.1 vs. 62.9 g SO2-eq, p < 0.001), lower eutrophication (high vs. low adherence: 21.9 vs. 23.6 g PO4-eq, p = 0.001) and lower land use (high vs*.* low adherence: 7.8 vs. 8.6 m2, p < 0.001).

In addition, to try to understand the relationship we obtained between greater adherence to MD and a greater amount of GHG emissions, we carried out an analysis eliminating fish and seafood intakes from the diet. In this case, in the model adjusted for total energy intake, all the environmental factors analysed decreased with greater adherence to MD. Higher adherence to MD was associated with lower GHG emissions (high vs. low adherence: 3035.3 vs. 3281.2 g CO2-eq, p < 0.001), lower acidification (high vs. low adherence: 54.8 vs. 65.4 g SO2-eq, p < 0.001), lower eutrophication (high vs. low adherence: 20.6 vs. 24.4 g PO4-eq, p < 0.001), lower land use (high vs. low adherence: 7.3 vs. 8.9 m2, p < 0.001) and lower energy use (high vs. low adherence: 9701.5 vs. 10,202.1 kJ, p = 0.006) (Supplementary material).

**Fig. 2:**
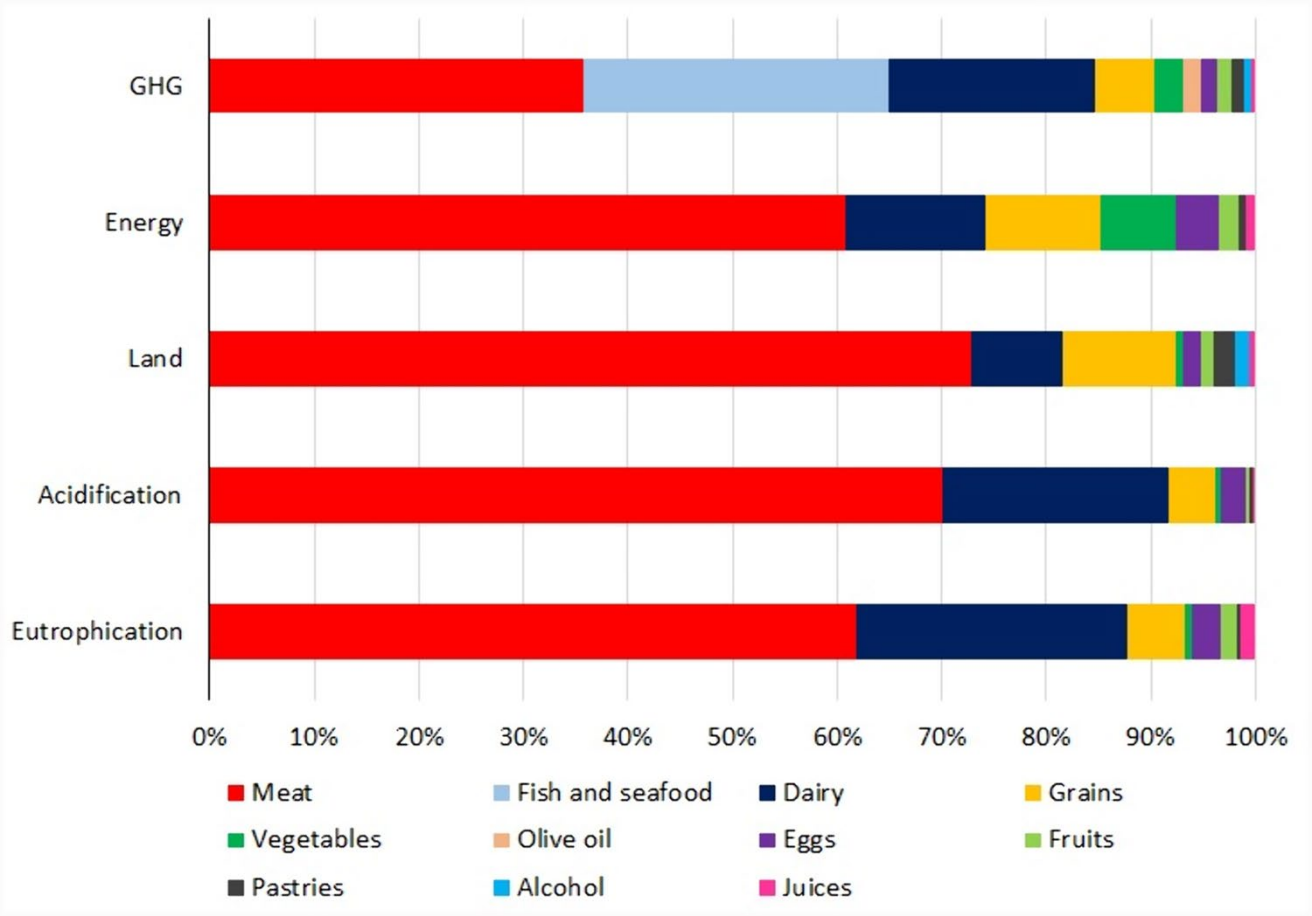
Percentage contribution of food groups to the different environmental factors analysed

The main contributor to GHG emissions was meat followed by fish and seafood (35.7% and 29.2% respectively); regarding energy use, the main contributor was meat followed by dairy (60.9% and 13.2% respectively); in the case of land use, the main contributor was meat followed by grains (72.8% and 11% respectively) and, finally, with respect to acidification and eutrophication, the main contributor was meat (70% and 61.8%) followed by dairy (21.8% and 25.9%) (Fig. 2).

## Discussion

The results of this work show that greater adherence to an MD pattern among participants in this cohort is associated with lower environmental impact in terms of acidification, eutrophication, and land use. In addition, the total energy intake-adjusted model also associated greater adherence to this dietary pattern with lower energy use. This is consistent with other published studies in which high adherence to MD was associated with lower energy and land use [30, 31]. In the study carried out by Fresán et al. [31] in which the actual diet consumed in a Mediterranean Spanish cohort was analysed, it was observed that greater adherence to the MD pattern was related to lower energy and land use in addition to improving other environmental factors.

In contrast to other studies [32, 33], our results indicate that greater adherence to MD is related to higher GHG emissions. This can be explained by the high consumption of fish (with high GHG values) of the participants, since Portugal is the third country in the world in which most fish is consumed behind Iceland and Japan [34]. In Portugal, the MD and the Atlantic Diet coexist and, although they have common characteristics, such as an abundant consumption of fruits and vegetables and the use of olive oil as the main fat, there is a greater consumption of fish, meat, legumes and potatoes in the Atlantic Diet [35].

Although the intake of fish is highly recommended from a nutritional point of view because of its wide-ranging health benefits, and consumption of at least two to three servings per week is recommended [36, 37], consuming more than this amount could be detrimental to the environment as it would increase the overexploitation to which fish stocks in more than 80% of the world are already subjected [38]. In addition, organisations such as the Council of Food Policy Advisors recommend a shift in consumption towards products sourced only from sustainable crops [39].

On the other hand, high fish consumption contributes to exposure and increased intake of heavy metals such as mercury [40]. Alternative with less environmental impact could be vegetable protein from legumes. As in the index proposed by Panagiotakos et al. [22], a higher consumption of fish is related to a greater adherence to the MD, we carried out a sensitivity analysis in which we eliminated these products from the calculations and obtained that, in this case, greater adherence to the MD also decreased GHG emissions.

In our work, we observed how meat products are responsible for a greater environmental impact in the five factors analysed. This is explained by the fact that most agricultural land is used by livestock, and this is the main cause of deforestation, loss of biodiversity and land degradation [41]. These data correspond to what is published in different articles relating to animal products, especially meat, with increases in GHG emissions and in land and energy use [4, 7, 42–44].

After meat, dairy is the food group with the second highest environmental impact in 4 of the 5 indicators used and is also the third product with the highest impact in the remaining indicator (GHG). This is in line with several published studies that establish that animal-based foods, especially meat and dairy, use far more resources and have a higher environmental impact than most plant-based products [45, 46].

Most of the papers that analyse the relationship between different dietary patterns and environmental impact agree that the shift towards diets with a lower animal product content and a higher consumption of plant products would be beneficial for the environment [2, 9, 47–49]. In the study published by Belgacem et al. [49], they show how the change of Western and European diets towards a MD pattern would mean less environmental impact in terms of GHG emissions, eutrophication, and land and water use. In addition, the revision published by Fresán et al. [45], they conclude that vegan and ovolactovegetarian diets generate lower GHG emissions and use fewer natural resources.

Other data obtained in the study were that participants with a greater adherence to the MD and therefore with a lower environmental impact on their diet were those with more years of schooling themselves and their parents, who performed physical exercise, non-smokers and who did not drink alcohol or did it less than once a week. Our results are partially consistent with those published by Sánchez-Villegas et al. [50], in which, in a Spanish cohort examining the relationship between sociodemographic factors and dietary patterns, non-smokers who were more physically active were in the highest quintile of adherence to MD. They are also consistent with various articles showing higher levels of education [51] or physical activity [52] are associated with greater adherence to the MD.

As the world's population is steadily increasing and is estimated to reach 10 billion by 2050 [53] and that food production is the main cause of global environmental change [4], it is essential that food guidelines and policies shift from a traditional health-only approach to a sustainability-sensitive approach [54], and, at this point, MD is presented as a possible solution to the health-diet-environment trilemma [55], as increasing adherence to this pattern, characterised by a low consumption of animal products, can help not only to improve our health, but also to reduce environmental impact as we have seen in our work.

### Strengths and limitations

The results presented in this paper are not without limitations. Since there is no single method for assessing the environmental impact of diet, the results may not be comparable quantitatively, and it should also be borne in mind that, within the database used, there are products that could be produced locally and therefore present different environmental impacts from that used for our calculations. In addition, the environmental impact database chosen did not include some of the foods included in the FFQ, and that this questionnaire included recipes that had to be broken down into main ingredients following standard recipes. Finally, the collection of intake data through this type of questionnaire could present a recall bias.

However, it also has numerous strengths. To our knowledge, this is the first time that the environmental impact of diet in Portugal has been analysed in association with MD. In addition, it was conducted in a large population sample who were recruited in schools, with a high proportion of participation (about 78%). Another strength is that this study is population-based, and its information may contribute to the planning and implementation of healthy food promotion strategies.

## Conclusions

Higher adherence to the MD is associated with lower environmental impact in terms of acidification, eutrophication, and land use, and even lower GHG emissions and lower energy use depending on the adjustment model used. Meat products have the most weight in terms of environmental impact in the five factors analysed, so it is expected that diets low in these products will be more environmentally sustainable.

Despite the impact of fish consumption on GHG emissions, our results support the recommendation of the Mediterranean Diet as a strategy to increase the health of the population and our planet.

## Supplementary Information

Below is the link to the electronic supplementary material.

Supplementary file1 (XLSX 21 KB)

Supplementary file2 (DOCX 127 KB)

## Data Availability

The data used and/or analyzed in the present study is available from the corresponding author on reasonable request.

## References

[1] Bjørnarå HB, Torstveit MK, Bere E (2019) Healthy and sustainable diet and physical activity: the rationale for and experiences from developing a combined summary score. Scand J Public Health 47(5):583–591. 10.1177/1403494818785056 29963968 10.1177/1403494818785056

[2] Aleksandrowicz L, Green R, Joy EJM et al (2016) The impacts of dietary change on greenhouse gas emissions, land use, water use, and health: a systematic review. PLoS One 11(11). 10.1371/journal.pone.0165797 10.1371/journal.pone.0165797 PMC5094759 27812156

[3] Myers SS, Smith MR, Guth S, et al (2017) Climate change and global food systems: potential impacts on food security and undernutrition. Ann Rev Pub Health 38:259–277. 10.1146/ANNUREV-PUBLHEALTH-031816-044356 10.1146/annurev-publhealth-031816-044356 28125383

[4] Tilman D, Clark M (2014) Global diets link environmental sustainability and human health. Nat 515:518–522. 10.1038/nature13959 10.1038/nature13959 25383533

[5] Clark MA, Domingo NGG, Colgan K et al (2020) Global food system emissions could preclude achieving the 1.5° and 2°C climate change targets. Science 370:705–708. 10.1126/SCIENCE.ABA7357 33154139 10.1126/SCIENCE.ABA7357

[6] Röös E, Sundberg C, Hansson P-A (2014) Carbon footprint of food products. Assessment of carbon footprint in different industrial sectors. Springer, Singapore, pp 85–112

[7] Hjorth T, Huseinovic E, Hallström E et al (2020) Changes in dietary carbon footprint over ten years relative to individual characteristics and food intake in the Västerbotten Intervention Programme. Sci Rep. 10.1038/S41598-019-56924-8 31913331 10.1038/S41598-019-56924-8 PMC6949226

[8] World Health Organization (2019) Sustainable healthy diets: guiding principles—food and agriculture organization of the United Nations. Italy, Rome

[9] Willett W, Rockström J, Loken B et al (2019) Food in the Anthropocene: the EAT–Lancet Commission on healthy diets from sustainable food systems. Lancet 393:447–492 30660336 10.1016/S0140-6736(18)31788-4

[10] Salas-Salvadó J, Mena-Sánchez G (2017) El gran ensayo de campo nutricional Predimed. Nutr Clin Med El. 10.7400/NCM.2017.11.1.5046 10.7400/NCM.2017.11.1.5046

[11] Martinez-Gonzalez MA, Bes-Rastrollo M (2014) Dietary patterns, Mediterranean diet, and cardiovascular disease. Curr Opin Lipidol 25:20–26. 10.1097/MOL.0000000000000044 24370845 10.1097/MOL.0000000000000044

[12] Álvarez-Álvarez I, Martínez-González MÁ, Sánchez-Tainta A et al (2018) Dieta mediterránea hipocalórica y factores de riesgo cardiovascular: análisis transversal de PREDIMED-Plus. Rev Española Cardiol. 10.1016/J.RECESP.2018.08.007 10.1016/J.RECESP.2018.08.007

[13] Guasch-Ferré M, Willett WC (2021) The Mediterranean diet and health: a comprehensive overview. J Intern Med 290:549–566. 10.1111/JOIM.13333 34423871 10.1111/JOIM.13333

[14] Salas-Salvado J, Bullo M, Babio N et al (2011) Reduction in the incidence of type 2 diabetes with the Mediterranean diet: results of the PREDIMED-Reus nutrition intervention randomized trial. Diabetes Care 34:14–19. 10.2337/dc10-1288 20929998 10.2337/dc10-1288 PMC3005482

[15] Serra-Majem L, Ortiz-Andrellucchi A (2018) La dieta mediterránea como ejemplo de una alimentación y nutrición sostenibles: enfoque multidisciplinar. Nutr Hosp 35. 10.20960/nh.2133 30070130 10.20960/nh.2133

[16] Germani A, Vitiello V, Giusti AM et al (2014) Environmental and economic sustainability of the Mediterranean diet. Int J Food Sci Nutr. 10.3109/09637486.2014.945152 25088933 10.3109/09637486.2014.945152

[17] Ramos E, Barros H (2007) Family and school determinants of overweight in 13-year-old Portuguese adolescents. Acta Pædiatrica 96:281–286. 10.1111/J.1651-2227.2007.00107.X 17429921 10.1111/J.1651-2227.2007.00107.X

[18] Willett WC, Howe GR, Kushi LH (1997) Adjustment for total energy intake in epidemiologic studies. Am J Clin Nutr 65(4 Suppl):1220S–1228S. 10.1093/ajcn/65.4.1220S 9094926 10.1093/ajcn/65.4.1220S

[19] Lopes C, Aro A, Azevedo A et al (2007) Intake and adipose tissue composition of fatty acids and risk of myocardial infarction in a male Portuguese community sample{a figure is presented}. J Am Diet Assoc 107:276–286. 10.1016/j.jada.2006.11.008 17258965 10.1016/j.jada.2006.11.008

[20] Willett WC, Sampson L, Stampfer MJ et al (1985) Reproducibility and validity of a semiquantitative food frequency questionnaire. Am J Epidemiol 122:51–65. 10.1093/OXFORDJOURNALS.AJE.A114086 4014201 10.1093/OXFORDJOURNALS.AJE.A114086

[21] Ferreira F, Graça M (1985) Composition table of Portuguese food, 2nd edn. National Institute of Health Dr Ricardo Jorge, Lisbon, Portugal

[22] Panagiotakos DB, Pitsavos C, Arvaniti F, Stefanadis C (2007) Adherence to the Mediterranean food pattern predicts the prevalence of hypertension, hypercholesterolemia, diabetes and obesity, among healthy adults; the accuracy of the MedDietScore. Prev Med (Baltim) 44:335–340. 10.1016/j.ypmed.2006.12.009 10.1016/j.ypmed.2006.12.009 17350085

[23] World Health Organization (2000) Obesity: preventing and managing the global epidemic. Report of a WHO consultation. World Heal Organ—Tech Rep Ser. 894:i–xii, 1–253 11234459

[24] Sánchez-Tainta A, San Julián B, Martínez-González MA, Miguel A (2015) PREDIMED, date el gusto de comer sano, 1st ed. EUNSA, Barañáin. ISBN: 978-84-313-3076-7

[25] Flo JM (1998) Postres del Mediterráneo. Ed. Planeta. ISBN: 978-84-08-02634-1

[26] Lopes C, Torres D, Oliveira A et al (2017) Inquérito Alimentar Nacional e de Atividade Física, IAN-AF 2015-2016: Relatório de resultados Autores

[27] Clark M, Tilman D (2017) Comparative analysis of environmental impacts of agricultural production systems, agricultural input efficiency, and food choice. Environ Res Lett 12:064016. 10.1088/1748-9326/AA6CD5 10.1088/1748-9326/AA6CD5

[28] R Core Team (2016). R: a language and environment for statistical computing. R Foundation for Statistical Computing, Vienna, Austria. https://www.R-project.org/

[29] StataCorp (2023) Stata statistical software: release 15. StataCorp LLC, College Station, TX

[30] Tepper S, Kissinger M, Avital K, Shahar DR (2022) The environmental footprint associated with the Mediterranean diet, EAT-Lancet diet, and the sustainable healthy diet index: a population-based study. Front Nutr 9:951. 10.3389/FNUT.2022.870883/BIBTEX 10.3389/FNUT.2022.870883/BIBTEX PMC9161357 35662947

[31] Fresán U, Martínez-Gonzalez MA, Sabaté J, Bes-Rastrollo M (2018) The Mediterranean diet, an environmentally friendly option: Evidence from the Seguimiento Universidad de Navarra (SUN) cohort. Public Health Nutr. 10.1017/S1368980017003986 29380717 10.1017/S1368980017003986 PMC10261578

[32] Naja F, Itani L, Hamade R et al (2019) Mediterranean diet and its environmental footprints amid nutrition transition: the case of Lebanon. Sustain. 10.3390/su11236690 10.3390/su11236690

[33] García S, Bouzas C, Mateos D et al (2023) Carbon dioxide (CO2) emissions and adherence to Mediterranean diet in an adult population: the Mediterranean diet index as a pollution level index. Environ Health 22:1. 10.1186/s12940-022-00956-7 36600281 10.1186/s12940-022-00956-7 PMC9814202

[34] Vaz-Velho ML, Pinheiro R, Rodrigues AS (2016) The Atlantic diet—origin and features. Int J Food Stud 5:106–119. 10.7455/ijfs/5.1.2016.a10 10.7455/ijfs/5.1.2016.a10

[35] Esteve-Llorens X, Darriba C, Moreira MT et al (2019) Towards an environmentally sustainable and healthy Atlantic dietary pattern: life cycle carbon footprint and nutritional quality. Sci Total Environ 646:704–715. 10.1016/J.SCITOTENV.2018.07.264 30059930 10.1016/J.SCITOTENV.2018.07.264

[36] Mohan D, Mente A, Dehghan M et al (2021) Associations of fish consumption with risk of cardiovascular disease and mortality among individuals with or without vascular disease from 58 countries. JAMA Intern Med 181:631. 10.1001/JAMAINTERNMED.2021.0036 33683310 10.1001/JAMAINTERNMED.2021.0036 PMC7941252

[37] Jurek J, Owczarek M, Godos J et al (2022) Fish and human health: an umbrella review of observational studies. Int J Food Sci Nutr 73:851–860. 10.1080/09637486.2022.2090520 35758202 10.1080/09637486.2022.2090520

[38] Thurstan RH, Brockington S, Roberts CM (2010) The effects of 118 years of industrial fishing on UK bottom trawl fisheries. Nat Commun 11(1):1–6. 10.1038/ncomms1013 10.1038/ncomms1013 20975682

[39] Clonan A, Holdsworth M, Swift JA et al (2012) The dilemma of healthy eating and environmental sustainability: the case of fish. Public Health Nutr 15:277–284. 10.1017/S1368980011000930 21619717 10.1017/S1368980011000930

[40] Castaño A, Cutanda F, Esteban M et al (2015) Fish consumption patterns and hair mercury levels in children and their mothers in 17 EU countries. Environ Res 141:58–68. 10.1016/J.ENVRES.2014.10.029 25667172 10.1016/J.ENVRES.2014.10.029

[41] Friel S, Dangour AD, Garnett T et al (2009) Public health benefits of strategies to reduce greenhouse-gas emissions: food and agriculture. Lancet 374:2016–2025. 10.1016/S0140-6736(09)61753-0 19942280 10.1016/S0140-6736(09)61753-0

[42] Sáez-Almendros S, Obrador B, Bach-Faig A, Serra-Majem L (2013) Environmental footprints of Mediterranean versus Western dietary patterns: beyond the health benefits of the Mediterranean diet. Environ Heal A Glob Access Sci Source. 10.1186/1476-069X-12-118 10.1186/1476-069X-12-118 PMC3895675 24378069

[43] Hedenus F, Wirsenius S, Johansson DJA (2014) The importance of reduced meat and dairy consumption for meeting stringent climate change targets. Clim Change 124:79–91. 10.1007/S10584-014-1104-5/FIGURES/3 10.1007/S10584-014-1104-5/FIGURES/3

[44] Rose D, Heller MC, Willits-Smith AM, Meyer RJ (2019) Carbon footprint of self-selected US diets: nutritional, demographic, and behavioral correlates. Am J Clin Nutr 109:526. 10.1093/AJCN/NQY327 30698631 10.1093/AJCN/NQY327 PMC6408204

[45] Fresán U, Sabaté J (2019) Vegetarian diets: planetary health and its alignment with human health. Adv Nutr 10:S380–S388 31728487 10.1093/advances/nmz019 PMC6855976

[46] Sabaté J, Sranacharoenpong K, Harwatt H et al (2015) The environmental cost of protein food choices. Public Health Nutr 18:2067–2073. 10.1017/S1368980014002377 25374332 10.1017/S1368980014002377 PMC10271869

[47] Davis KF, Gephart JA, Emery KA et al (2016) Meeting future food demand with current agricultural resources. Glob Environ Chang 39:125–132. 10.1016/J.GLOENVCHA.2016.05.004 10.1016/J.GLOENVCHA.2016.05.004

[48] Berardy AJ, Rubín-García M, Sabaté J (2022) A scoping review of the environmental impacts and nutrient composition of plant-based milks. Adv Nutr. 10.1093/advances/nmac098 36083996 10.1093/advances/nmac098 PMC9930689

[49] Belgacem W, Mattas K, Arampatzis G, Baourakis G (2021) Changing dietary behavior for better biodiversity preservation: a preliminary study. Nutrients. 10.3390/nu13062076 34204478 10.3390/nu13062076 PMC8234216

[50] Sánchez-Villegas A, Delgado-Rodríguez M, Martínez-González MÁ et al (2003) Gender, age, socio-demographic and lifestyle factors associated with major dietary patterns in the Spanish Project SUN (Seguimiento Universidad de Navarra. Eur J Clin Nutr 572(57):285–292. 10.1038/sj.ejcn.1601528 10.1038/sj.ejcn.1601528 12571661

[51] Onetti W, Álvarez-Kurogi L, Castillo-Rodríguez A, et al (2019) Adherencia al patrón de dieta mediterránea y autoconcepto en adolescentes. Nutr Hosp 36:658–664. 10.20960/NH.02214 10.20960/nh.02214 30985186

[52] Tárraga Marcos A, Panisello Royo JM, Carbayo Herencia JA, et al (2021) Valoración de la adherencia a la dieta mediterránea en estudiantes universitarios de Ciencias de la Salud y su relación con el nivel de actividad física. Nutr Hosp 38:814–820. 10.20960/NH.03531 10.20960/nh.03531 34024112

[53] FAO (2018) Transformar la alimentación y la agricultura para alcanzar los ODS—20 acciones interconectadas para guiar a los encargados de adoptar decisiones. Organ las Nac Unidas para la Aliment y la Agric 76

[54] Berry EM, Dernini S, Burlingame B et al (2015) Food security and sustainability: can one exist without the other? Public Health Nutr 18:2293–2302. 10.1017/S136898001500021X 25684016 10.1017/S136898001500021X PMC10271846

[55] Dernini S, Berry EM (2015) Mediterranean diet: from a healthy diet to a sustainable dietary pattern. Front Nutr. 10.3389/fnut.2015.00015 26284249 10.3389/fnut.2015.00015 PMC4518218

