# Supplementary material for: Environmental impact of the diet of young Portuguese and its relationship with adherence to the Mediterranean Diet
Source: Eur J Nutr. 2024 May 19;63(6):2307–15. doi: 10.1007/s00394-024-03396-w (PMC11377495; doi:10.1007/s00394-024-03396-w)
Supplement: Supplementary file 2 — Supplementary file2 (DOCX 127 KB) [file 394_2024_3396_MOESM2_ESM.docx]

**Tittle:** Environmental impact of the diet of young Portuguese and its relationship with adherence to the Mediterranean Diet.

**Journal:** European Journal of Nutrition

**Authors:** Laura Álvarez-Álvarez et al.

**Corresponding author:** Elisabete Ramos; up426486@g.uporto.pt

**Supplementary material**

**Table 3**. Environmental footprint of different factors by tertiles of the Diet Score (DS) according to different adjustment models without fish and seafood.

|  | Linear regression model adjusted for total energy intake (excluding fish and seafood) | | | | | | | | | |  |
| --- | --- | --- | --- | --- | --- | --- | --- | --- | --- | --- | --- |
|  | Low adherence to the MD | | Medium adherence to the MD | | | High adherence to the MD | | | *p-value* |  | |
|  | Mean | IC 95% | | Mean | IC 95% | | Mean | IC 95% |  | | |
| GHG (g CO2-eq) | 3281.2 | 74.1272 | | 3283.37 | 74.774 | | 3035.29 | 79.086 | **<0.001** | | |
| ACIDIFICATION (g SO2-eq) | 65.4 | 1.6856 | | 62.67 | 1.7052 | | 54.79 | 1.7836 | **<0.001** | | |
| EUTROPHICATION (g PO4-eq) | 24.36 | 0.6272 | | 23.62 | 0.6272 | | 20.56 | 0.6664 | **<0.001** | | |
| LAND (m^2^) | 8.87 | 0.2744 | | 8.43 | 0.2744 | | 7.34 | 0.2744 | **<0.001** | | |
| ENERGY (kJ) | 10202.05 | 240.1588 | | 10463.27 | 242.2364 | | 9701.46 | 256.2112 | **<0.001** | | |

Linear regression models adjusted for total energy intake were performed to classify participants based on tertiles of adherence to the MD excluding fish and seafood intakes. Kruskal-Wallis tests were used to assess differences between tertiles with respect to GHG emissions, land and energy use, acidification, and eutrophication.

*MD indicates Mediterranean Diet; GHG, greenhouse gas emissions; CI, Confidence Interval; g CO2-eq, grams of Carbon Dioxide equivalents; g SO2-eq, grams of Sulfur Dioxide equivalents; g PO4-eq, grams of Phosphate equivalents; and kJ, kilojoules.

The results highlighted in bold are those statistically significant (p<0.05).

**Fig. 3** Environmental footprint for different factors per tertiles of adherence to DM without regard to fish**
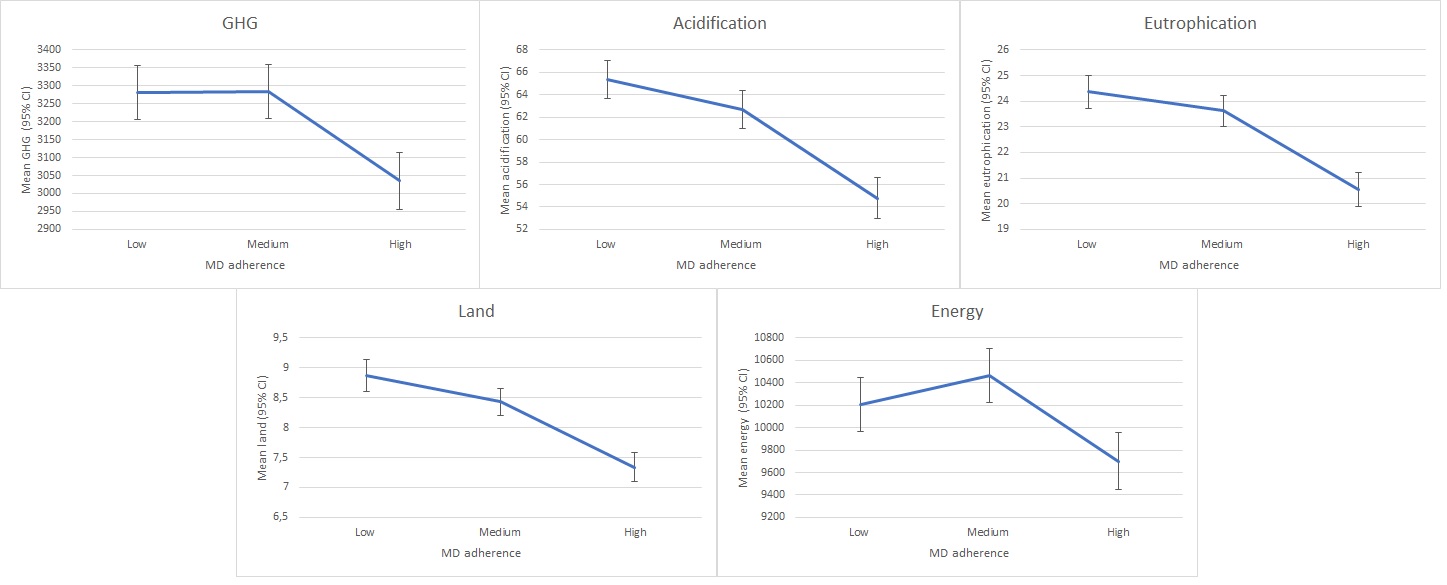
**

**Figure 3.** Linear regression models adjusted for total energy intake were performed to classify participants based on tertiles of adherence to MD excluding fish and seafood intakes. Kruskal-Wallis tests were used to assess differences between tertiles with respect to GHG emissions, land and energy use, acidification, and eutrophication.

GHG indicates Greenhouse gas emissions, and CI, Confidence Interval. GHG is expressed as grams of Carbon Dioxide equivalents; acidification, as grams of Sulfur Dioxide equivalents; eutrophication, as grams of Phosphate equivalents; land, as m^2^; and energy, as kilojoules.
